# Beaver in the liver

**DOI:** 10.11604/pamj.2017.27.138.12227

**Published:** 2017-06-23

**Authors:** Mohamad Syafeeq Faeez Md Noh, Siti Jusna @ Siti Sharifah Muhammad

**Affiliations:** 1 Department of Imaging, Faculty of Medicine and Health Sciences, Universiti Putra, Malaysia; 2 Department of Diagnostic Imaging, Hospital Serdang, Malaysia

**Keywords:** Computed tomography, elongated left liver lobe, ultrasonography

## Image in medicine

A 60-year-old woman with a congenital solitary right kidney presented with 1 month history of right flank pain associated with hematuria and urinary tract infection (UTI) symptoms. A Computed tomography (CT) was done in view of the precious kidney, and revealed features suggestive of right obstructive uropathy with pyelonephritis; for which appropriate treatment was subsequently instituted. Incidentally, an elongated left liver lobe was discovered. This anatomical variant, also known as 'Beaver tail liver' is seen to be extending beyond the spleen, almost encasing it entirely (A). The term was coined due to its resemblance to the tail of a beaver (B). Although not widely reported in the medical literature, the presence of this variant has a few clinical implications. When present, lesions occurring in the elongated left lobe may be missed on ultrasonography (USG). In cases of trauma, injury to the left side of the abdomen which classically affects the spleen, may affect the elongated left liver lobe. It may also be mistaken for a splenic hematoma. Operative intervention, when pursued without prior knowledge of its presence, may result in unwanted complications. In essence, clinicians should be aware of its occurrence and clinical significance; as this may influence patient management and treatment outcome.

**Figure 1:**
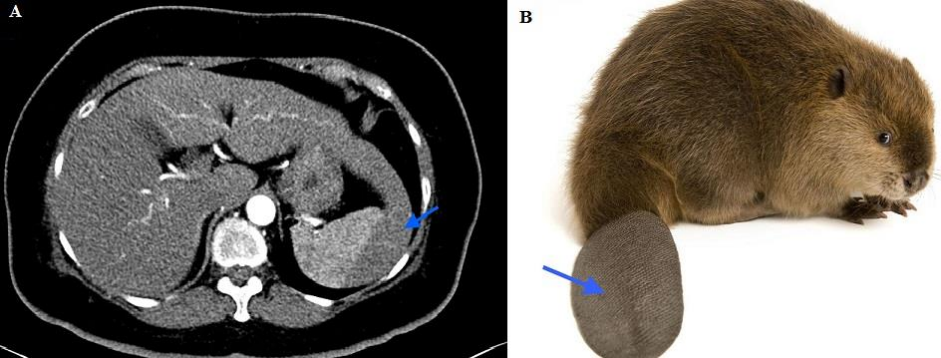
(A) abdominal CT (Computed tomography) in axial section, showing the ‘beaver tail liver’ (arrow) otherwise known as an elongated left lobe of the liver; this is seen extending beyond the spleen, almost encasing it entirely; (B) note the resemblance of the tail of a beaver (arrow) to the appearance of the elongated left liver lobe (A)

